# Hypopituitarism Presenting as Recurrent Episodes of Hypoglycemia: Houssay Phenomenon

**DOI:** 10.7759/cureus.37530

**Published:** 2023-04-13

**Authors:** Tehlil Rizwan, Gunjan Arora, Marwah Alchalabi, Faisal Qureshi

**Affiliations:** 1 Internal Medicine, Ascension St. Joseph Hospital, Chicago, USA; 2 Endocrinology, Diabetes, and Metabolism, Ascension St. Joseph Hospital, Chicago, USA

**Keywords:** endocrine emergency, rare cause of altered mental status, recurrent hypoglycemia, hypopituitarism, secondary adrenal insufficiency

## Abstract

Hypopituitarism, a rare disorder, is defined as decreased production and secretion of one or more of the hormones that are normally secreted by the pituitary gland, resulting from the diseases of the pituitary gland itself or the hypothalamus. The clinical manifestations of this disorder are usually nonspecific and can lead to life-threatening complications and mortality. Here, we present a case of a 66-year-old female patient who was brought to the ER by her family with concerns of altered mentation. The altered mentation was found to be secondary to a severe hypoglycemic episode, which was later discovered to be due to underlying panhypopituitarism with secondary adrenal insufficiency. Endocrinology was consulted and recommended assessment of the hypothalamic-pituitary axis. The tests revealed low levels of serum insulin and C-peptide along with decreased levels of luteinizing hormone (LH), follicle-stimulating hormone (FSH), prolactin, cortisol, free thyroxine (T4), and adrenocorticotropic hormone (ACTH). She was started on intravenous hydrocortisone and levothyroxine, which were later switched to oral hydrocortisone and levothyroxine after the stabilization of her blood glucose levels. She was later advised to follow up with endocrinology upon discharge. While evaluating a patient with hypoglycemia, it is important to keep hypopituitarism causing secondary adrenal insufficiency in mind as a differential diagnosis because it can be life-threatening if not recognized early and treated in a timely manner.

## Introduction

Hypopituitarism is defined as decreased production and secretion of one or more of the hormones that are normally secreted by the pituitary gland, resulting from the diseases of the pituitary gland itself or the hypothalamus. Hypopituitarism is a rare disease. As demonstrated in a study from Spain, the incidence and prevalence are estimated to be 4.2 per 100,000 per year and 45.5 per 100,000, respectively [1]. There are less than 200,000 cases of hypopituitarism in the United States [2]. Tanriverdi et al. [3] classified the causes of hypopituitarism as pituitary tumors (46.6%), non-tumoral (approximately 50%), and extra-pituitary tumors (7.2%).

The clinical manifestations of this disorder are usually nonspecific; however, they can lead to life-threatening complications and mortality [4]. The signs and symptoms are usually related to the type and degree of hormone insufficiency. Patients may be asymptomatic or present with symptoms such as fatigue, nausea, vomiting, dizziness, and depression. In addition, tumoral masses can present with mass effects (headache and nausea), visual field defects, and neurological deficits.

Here, we present a case of a 66-year-old female patient who was brought to the emergency room (ER) by her family with concerns of altered mentation, found to be secondary to a severe hypoglycemic episode. It was later discovered to be due to underlying panhypopituitarism with secondary adrenal insufficiency. The rarity of the disease along with the clinical presentation of the patient - recurrent episodes of hypoglycemia coinciding with periods of acute stress in the background of fatigue, decreased appetite, and weight loss - makes this a compelling case to report.

## Case presentation

A 66-year-old female with a medical history significant for diabetes, hypertension, hyperlipidemia, stage 3 chronic kidney disease, osteoarthritis s/p right-sided total knee arthroplasty (18 months prior to current admission), gastroesophageal reflux disease, and anemia was brought to the emergency room (ER) by her family due to acute onset confusion with agitation. The patient was in her usual state of health four days prior to admission when she developed non-bloody diarrhea and decreased oral intake. She was noted to be confused, forgetful, and combative on the morning of the admission. She had no known history of alcohol or drug use. On presentation to the ER, she was hemodynamically stable. On physical examination, she was alert and oriented to her name only, slightly agitated, but was calm and able to be redirectable. She was found to have decreased range of motion in her right lower extremity due to her recent history of knee replacement surgery. The rest of her examination was unremarkable. Initial laboratory evaluation revealed glucose at 20 mg/dl, which improved to 119 mg/dl after administration of 50 ml of dextrose 50%. Other labs included WBC count at 3.6 k/mm cu, hemoglobin at 10.5 g/dL, platelets at 279 k/mm cu, creatinine at 1.25 mg/dL, blood urea nitrogen (BUN) at 23 mg/dL, sodium at 139 mmol/L, potassium at 3.5 mmol/L, and lactic acid at 1.5 mmol/L. EKG showed normal sinus rhythm. The CT of the head was unremarkable. Her mental status improved after the correction of hypoglycemia.

She endorsed similar episodes of hypoglycemia several months prior to this presentation and after undergoing right knee arthroplasty. She also reported weight loss. Chart review revealed significant weight loss of 38 pounds over the past 12 months. Of note, she had a history of diabetes for which she was prescribed metformin in the past. However, when periodic glycosylated hemoglobin (HbA1c) and glucose levels normalized, she was taken off metformin.

Upon admission to telemetry, endocrinology was consulted. She was started on dextrose 5% in normal saline at 100 ml/hour along with point-of-care glucose monitoring and hypoglycemia protocol. Overnight, she became hypoglycemic again to 35 mg/dl, which improved to 107 mg/dl after she received 25 grams of dextrose 50%. Serum insulin and C-peptide levels were tested and were found to be 0.2 uIU/mL (normal = 1.9-23 uIU/mL) and 0.10 ng/mL (normal = 0.5-3.3 ng/ml), respectively. The serum insulin and C-peptide levels ruled out exogenous insulin use and insulinoma, which then warranted assessment of the hypothalamic-pituitary axis. The tests revealed low levels of serum insulin and C-peptide along with decreased levels of luteinizing hormone (LH), follicle-stimulating hormone (FSH), prolactin, cortisol, free thyroxine (T4), and adrenocorticotropic hormone (ACTH).

The next evening, she became hypoglycemic again to 42 mg/dL despite being on a 5% dextrose normal saline infusion. Thyroid-stimulating hormone (TSH) was in the lower-normal limits. Prolactin, LH, FSH, and cortisol came back as low, and free T4 was undetectable. The laboratory investigations are summarized in Table 1.

**Table 1:** The laboratory investigations revealed low levels of serum insulin and C-peptide along with decreased levels of LH, FSH, prolactin, cortisol, free T4, and ACTH.

No.	Assay	Result	Normal range
1.	Serum insulin	0.2 uIU/ml (low)	1.9-23 uIU/ml
2.	C-peptide	0.10 ng/ml (low)	0.5-3.3 ng/ml
3.	Luteinizing hormone (LH)	0.31 mIU/ml (low)	10.87-58.64 mIU/ml (post-menopause)
4.	Follicle-stimulating hormone (FSH)	0.23 mIU/ml (low)	16.74-113.59 mIU/ml (post-menopause)
5.	Prolactin	0.5 ng/ml (low)	3.34-26.72 ng/ml
6.	Cortisol	1 mcg/dl (low)	6.2-19.4 mcg/dl
7.	Thyroid-stimulating hormone (TSH)	0.393 uIU/ml	0.27-4.2 uIU/ml
8.	Free thyroxine (T4)	<0.25 ng/dl (low)	0.5-1.6 ng/dl
9.	Adrenocorticotropic hormone (ACTH)	<1.5 pg/ml (low)	7.2-63.3 pg/ml

**Figure 1:**
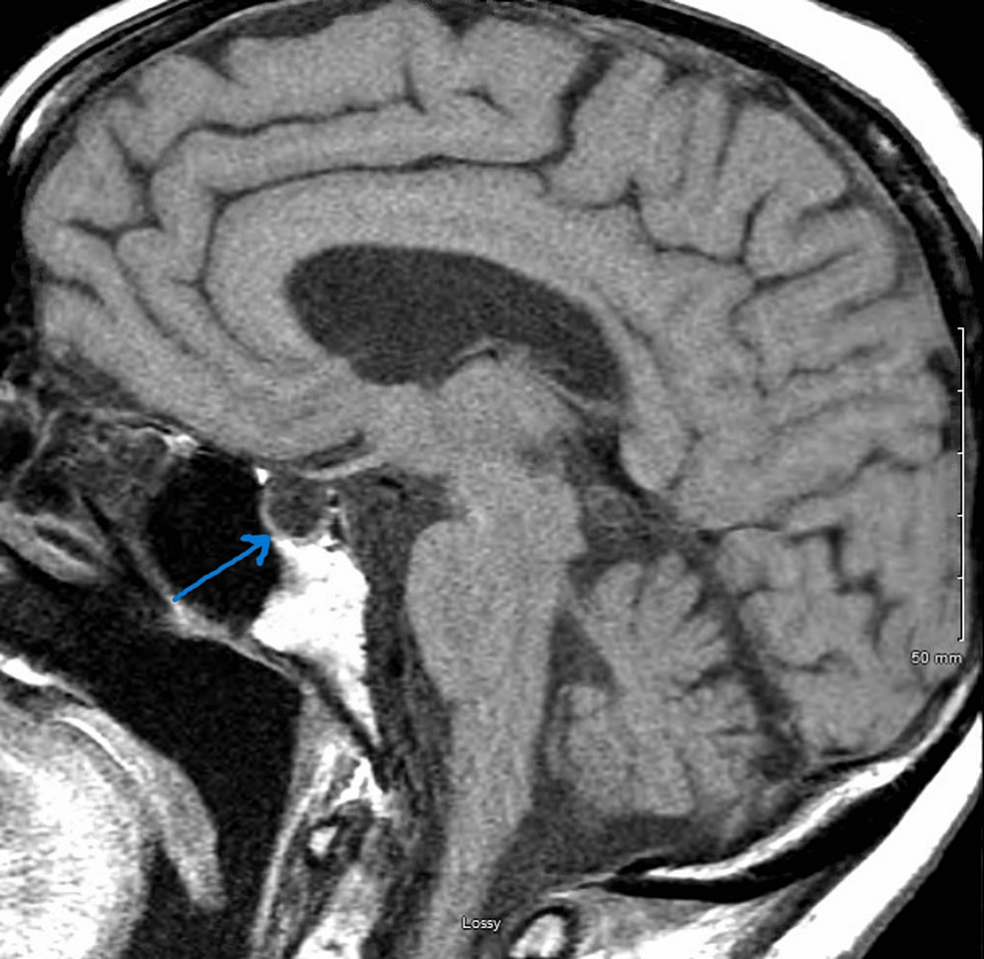
MRI showing empty sella.

Endocrinology recommended administering IV hydrocortisone 100 mg every six hours while waiting for the results of the ACTH stimulation test. They also recommended one dose of IV levothyroxine 100 mcg after administration of the first dose of IV hydrocortisone. MRI of the brain with a pituitary focus was obtained and showed a slightly expanded, partially empty sella as illustrated in Figure 1.

A cosyntropin stimulation test was done and results included ACTH at baseline = 1.5, cortisol at 30 minutes = 2.8, and cortisol at one hour = 3. The dose of IV hydrocortisone was decreased from 100 mg every six hours to IV hydrocortisone 100 mg every 12 hours. Her blood glucose levels remained within normal limits, and she was discharged on a twice-daily regimen of hydrocortisone (20 mg in the morning and 10 mg at night). She was also advised levothyroxine 125 mcg daily. The patient followed up with endocrinology six months after her discharge. She is currently on a stable dose of hydrocortisone and levothyroxine. Further, endocrinology recommended adjusting doses of levothyroxine based on free T4 instead of TSH in the future due to inappropriate negative feedback mechanisms in the setting of central hypothyroidism.

## Discussion

Hypoglycemia is an endocrine emergency that should not only be treated aggressively but also warrants a thorough evaluation. In a previously diabetic patient, the most common culprits are medication overuse, increased physical activity, and decreased oral intake [5]. After these have been excluded, attention should be brought to other potential causes, including hepatic/renal failure, sepsis, excessive alcohol intake, non-islet cell tumors, insulinoma, insulin autoimmune hypoglycemia, and hypopituitarism leading to loss of counter-regulatory mechanisms [6].

Hypopituitarism is a rare cause of secondary adrenal insufficiency and thus can lead to hypoglycemic episodes due to the loss of counter-regulatory mechanisms. Hypopituitarism often presents with signs and symptoms related to decreased hormonal concentration. It can be associated with recurrent episodes of hypoglycemia, especially in the setting of acute stress/illness/surgery. Our patient had a history of multiple accounts of hypoglycemia associated with an acute stressor - she had an episode of hypoglycemia immediately following the right total knee replacement.

The phenomenon of recurrent hypoglycemic episodes in a diabetic patient due to hypopituitarism has been reported previously in various case reports. Hypoglycemia and sometimes resolution of diabetes have been shown in patients with anterior pituitary dysfunction due to loss of counter-regulatory mechanisms, which as a result leads to increased insulin sensitivity predisposing to severe hypoglycemia [7-9]. This phenomenon is named as Houssay phenomenon after Bernardo Houssay based on his experiments in 1931 and his explanation of amelioration of diabetes in dogs that had undergone pancreatectomy following the loss of counter-regulatory hormones by removal of the anterior pituitary gland [10].

Hypopituitarism, although rare, is an important cause of recurrent hypoglycemia driven by secondary adrenal insufficiency. Secondary adrenal insufficiency is fatal if not recognized and treated in a timely manner with steroid therapy. Hydrocortisone is the treatment of choice. Our patient improved significantly without having further episodes of hypoglycemia. She is following up with her primary care physician and endocrinologist.

## Conclusions

Hypoglycemia is an endocrine emergency and should be treated aggressively. In a previously diabetic patient, the potential causes of hypoglycemia include medication overuse, increased physical activity, and decreased oral intake. While evaluating a patient with hypoglycemia, it is important to keep hypopituitarism causing secondary adrenal insufficiency in mind as a differential diagnosis because it can be life-threatening if not recognized early and treated in a timely manner.
